# Endogenous thrombin potential following hemostatic therapy with 4-factor prothrombin complex concentrate: a 7-day observational study of trauma patients

**DOI:** 10.1186/cc13982

**Published:** 2014-07-09

**Authors:** Herbert Schöchl, Wolfgang Voelckel, Marc Maegele, Lukas Kirchmair, Christoph J Schlimp

**Affiliations:** 1 Department of Anaesthesiology and Intensive Care Medicine, AUVA Trauma Centre Salzburg, Franz Rehrl Platz 5, 5020, Salzburg, Austria; 2 Ludwig Boltzmann Institute for Experimental and Clinical Traumatology, AUVA Research Centre, Vienna, Austria; 3 Department of Trauma and Orthopedic Surgery, University of Witten/Herdecke, Cologne-Merheim Medical Center (CMMC), Cologne, Germany

## Abstract

**Introduction:**

Purified prothrombin complex concentrate (PCC) is increasingly used as hemostatic therapy for trauma-induced coagulopathy (TIC). However, the impact of PCC administration on coagulation status among patients with TIC has not been adequately investigated.

**Methods:**

In this observational, descriptive study, data relating to thrombin generation were obtained from plasma samples gathered prospectively from trauma patients upon emergency room (ER) admission and over the following 7 days. Standard coagulation tests, including measurement of antithrombin (AT) and fibrinogen, were performed. Three groups were investigated: patients receiving no coagulation therapy (NCT group), patients receiving fibrinogen concentrate only (FC group), and patients treated with PCC and fibrinogen concentrate (FC-PCC group).

**Results:**

The study population (77 patients) was predominantly male (84.4%); mean age was 40 ± 15 years and mean injury severity score was 25.6 ± 12.7. There were no significant differences between the three study groups in thrombin-related parameters upon ER admission. Endogenous thrombin potential (ETP) was significantly higher in the FC-PCC group compared with the NCT group on days 1 to 4 and the FC group on days 1 to 3. AT levels were significantly lower in the FC-PCC group from admission until day 3 (versus FC group) or day 4 (versus NCT group). Fibrinogen increased over time, with no significant between-group differences after ER admission. Despite ETP being higher, prothrombin time and activated partial thromboplastin time were significantly prolonged in the FC-PCC group from admission until day 3 to 4.

**Conclusions:**

Treatment with PCC increased ETP for several days, and patients receiving PCC therapy had low AT concentrations. These findings imply a potential pro-thrombotic state not reflected by standard coagulation tests. This is probably important given the postoperative acute phase increase in fibrinogen levels, although studies with clinical endpoints are needed to ascertain the implications for patient outcomes. We recommend careful use of PCC among trauma patients, with monitoring and potentially supplementation of AT.

## Introduction

Approximately one quarter to one third of all trauma patients present with coagulopathy detectable by standard coagulation tests such as prothrombin time (PT) or activated partial thromboplastin time (aPTT) [1,2]. Early and aggressive coagulation therapy is proven to be beneficial in these patients [3,4]. In most trauma centers worldwide fresh frozen plasma (FFP) is used first line to increase hemostatic capacity [4]. FFP contains both coagulation factors and inhibitors, but the thawing process often results in a substantial time delay before treatment can be started and only high-volume trauma centers store pre-thawed plasma for immediate use [5-7]. Concentrations of coagulation factors in FFP are determined by the donor levels, meaning considerable variability between units [8]. Moreover, physiological levels limit the extent to which patients’ coagulation factor levels can be raised by FFP [9].

Coagulation factor concentrates such as purified human fibrinogen concentrate and prothrombin complex concentrate (PCC) are considered as potential alternatives to FFP [10,11]. These substances are immediately available and contain well-defined quantities of coagulation proteins. Coagulation management based on infusion of concentrates under guidance from point-of-care coagulation monitoring (thrombelastography or thromboelastometry) has been proposed [12]. Fibrinogen concentrate is administered first line to correct low levels of fibrinogen, which are rapidly detectable by impaired fibrin-based clot formation (FIBTEM assay or functional fibrinogen assay) [13-16]. Among patients with adequate fibrinogen levels but persistent bleeding and prolonged initiation of coagulation (rapidly detectable by prolonged clotting time), PCC may be administered to increase thrombin generation (TG) [15]. However, there is little evidence to support PCC use in trauma-induced coagulopathy (TIC) [17,18].

Trauma studies in animals have revealed reduced blood loss and improved survival following PCC administration, in comparison with placebo [19-22]. Small clinical reports have described favorable outcomes when using PCC either alone or in combination with fibrinogen concentrate in trauma patients [15,17,23-26]. However, safety data following PCC administration unrelated to reversal of vitamin K antagonists are lacking. In a porcine multiple trauma model, Grottke *et al*. reported disseminated intravascular coagulation in four out of nine animals receiving 50 IU/kg bodyweight of PCC [27].

Trauma patients are prone to thromboembolic complications regardless of hemostatic therapy [28,29], and TG parameters have not been studied following PCC administration. We hypothesized that PCC increases thrombin potential in patients with severe bleeding trauma. We analyzed blood samples from trauma patients admitted to the AUVA trauma center Salzburg with the aim of assessing TG parameters upon emergency room (ER) admission and over the following 7 days. Three groups of patients were compared: those receiving no coagulation therapy (NCT group), those treated with fibrinogen concentrate only (FC group) and those who received PCC in combination with fibrinogen concentrate (FC-PCC group).

## Materials and methods

This prospective, observational, descriptive cohort study was performed on plasma samples gathered prospectively from trauma patients admitted to the AUVA Trauma Center Salzburg, Austria between March 2010 and November 2011. The inclusion criterion was admission to the ER following full trauma-team activation. The exclusion criteria were age <18 years, burns, pregnancy and known coagulation disorders. Informed consent was obtained from the patients or their relatives. The study was approved by the local ethics committee (E-1231 Votum), Sebastian-Stief-Gasse 2, 5020, Salzburg, Austria, with reference number EK-1127-2009.

### Coagulation therapy (algorithm-based)

The study population was separated into three cohorts according to the coagulation therapy received: trauma patients who received no coagulation therapy (NCT group), patients treated with fibrinogen concentrate only (FC group), and patients who received both fibrinogen concentrate and PCC (FC-PCC group). Coagulation management was based on our institutional protocol for patients with bleeding trauma, with treatment determined by viscoelastic coagulation tests (ROTEM®, Tem International, Munich, Germany) [30]. Briefly, ROTEM analyses are performed upon ER admission, during initial operative treatment and following ICU admission. Fibrinogen concentrate (Haemocomplettan P, CSL Behring, Marburg, Germany) is considered as first-line therapy in patients with impaired fibrin polymerization, that is, a clot amplitude (CA) of <7 mm after 10 minutes running time in the extrinsically activated test plus cytochalasin D (FIBTEM) assay (target FIBTEM CA10: 10 to 12 mm). If clotting time (CT) in the extrinsically activated assay (EXTEM) remains prolonged (>80 sec) following fibrinogen concentrate treatment, PCC (Prothromplex Total 600 IE, Baxter, Vienna, Austria) is administered. For patients with obviously severe coagulopathy upon admission, immediate treatment with both fibrinogen concentrate (6 to 8 g) and PCC (20 to 30 IU/kg bodyweight) is administered. Like other PCCs, Prothromplex Total is standardized according to FIX, with each vial containing 600 IU of this coagulation factor [31]. The dose of FII, less well defined by the package insert, has been reported as 750 IU per vial [31]. Tranexamic acid (TXA; 15 to 20 mg/kg bodyweight for patients with injury severity score (ISS) >16 and/or severe shock) was incorporated into our treatment protocol in 2011 following publication of the CRASH-2 data [32]. ISS is routinely calculated early at our center, using results from a computed tomography scan performed within 15 minutes of admission (the potential benefits of early scanning have been reported separately [33]).

The standard protocol recommends blood sampling as soon as possible following ER admission. In severely injured patients, blood is drawn after placement of an arterial line, whereas in less severe trauma where immediate invasive blood pressure monitoring is not considered necessary, blood is collected from venous lines. Blood samples are also drawn every morning for full blood cell count and standard coagulation tests until day 7 following ER admission. Thus, the only procedure in this study comprising an addition to routine monitoring was the measurement of TG parameters (using residual plasma specimens from samples that were collected for routine tests).

Demographic data, laboratory data, trauma severity scores such as ISS, Glasgow coma scale (GCS) score and outcome data were obtained from the electronic trauma database. In all cases, the attending physician was free to treat patients at his/her own discretion.

### Standard coagulation tests

Citrated blood samples were centrifuged and the following coagulation parameters were assessed: fibrinogen concentration (Clauss method: electromechanical detection using an STA-Compact analyzer (Diagnostics Stago ASA, Asnieres sur Seine, France); normal range 200 to 450 mg/dL), PT (measured using Sysmex XE-2100, Roche Diagnostics, Mannheim, Germany; normal range 11 to 16.1 sec), aPTT (measured using Sysmex XE-2100, Roche Diagnostics; normal range 23.7 to 34.9 sec ) and antithrombin (AT) (normal range 80 to 120%).

### Thrombin-related measurements

Citrated blood samples were centrifuged twice at 3,200 *g* for 20 minutes, and samples of platelet-poor plasma (PPP) were frozen at −80°C until analysis. TG was stimulated by tissue factor and measured using the Calibrated Automated Thrombogram (CAT; Thrombinoscope BV, Maastricht, The Netherlands).

The PPP samples were thawed in a water bath at 37°C, and centrifuged for 5 minutes at 10,000 *g* at room temperature. The measurements were performed in duplicate using 96-well plastic plates (Immulon 2HB clear 96-well, Thermo Electron, Boston, MA, USA), and all reagents were pre-warmed to 37°C: 20 μl of PPP-Reagent (Thrombinoscope BV) and 80 μl PPP were added to each well manually. After a brief incubation, 20 μl of thrombin substrate and calcium chloride (Fluo-Substrate and Fluo-Buffer, Thrombinoscope BV) were added automatically. The final concentration in the well was 5 pM for tissue factor and 4 μM phospholipids. A Fluoroskan Ascent fluorometer (Thermo Scientific, Waltham, MA, USA) was used to record continuous generation of thrombin. Each well was calibrated to a parallel well with a thrombin calibrator (Thrombin calibrator TS 20.0, Thrombinoscope BV).

The following parameters were recorded: lag time, peak thrombin generation, time to peak, and endogenous thrombin potential (ETP). Lag time (sec) was defined as the period between the addition of calcium and substrate reagent and the first active thrombin concentration higher than 10 nmol/L. Peak thrombin generation (nM) was defined as the highest active thrombin concentration during 90 minutes of monitoring. The time to peak was defined as the period from the start of the reaction until peak thrombin generation. ETP (area under the curve (AUC), nM.minutes) was the area under the thrombin concentration versus time graph, from 1 to 90 minutes.

### Statistical analysis

For all parameters, normality of the data distribution was tested using the Kolmogorov-Smirnov test. Normally distributed results were expressed as mean ± standard deviation, and those distributed otherwise were expressed as median and interquartile range (IQR) (25^th^ percentile, 75^th^ percentile). Between-group differences were analyzed using Student *t*-test, Mann-Whitney rank sum test, Newman-Keuls test or Kruskal-Wallis test with Dunn’s multiple comparison as appropriate. Within-group changes over time in ETP and AT levels were assessed using the Newman-Keuls test. Statistical calculations were performed using GraphPad Prism 5.03 (GraphPad Software, La Jolla, CA, USA). The level of significance was set at *P* <0.05.

## Results

The study population comprised 77 patients, 65 male and 12 female, with a mean age of 40 ± 15 years. The mean ISS was 25.6 ± 12.7 and the median GCS was 14 (8 to 15). All patients survived until hospital discharge. No coagulation therapy was considered necessary in 37 patients (NCT group); 23 patients were treated with fibrinogen concentrate only (FC-group) and 17 patients received PCC and fibrinogen concentrate (FC-PCC group). Demographic and clinical data on admission to the ER are shown in Table 1. ISS was significantly higher in the FC-PCC group than in the NCT group (*P* <0.0001).

**Table 1:** Clinical and demographic data upon emergency room admission

	**NCT group**	**FC group**	**FC-PCC group**	***P*-values**	
				**FC-PCC versus NCT**	**FC-PCC versus FC**
ISS	18.8 ± 9.4	29.0 ± 11.0	35.7 ± 13.0	<0.0001	ns
Age, years	46 ± 17	40 ± 14	36 ± 13	0.028	ns
Hb, g/dL	12.8 ± 2.2	12.6 ± 2.0	10.1 ± 2.6	0.0002	0.002
Platelet count × 10^3^/μL	225 ± 59	210 ± 54	191 ± 39	0.0334	ns
PT, sec	13.7 (12.7 to 14.6)	14.6 (13.8 to 15.2)	17.2 ± 3.1	0.0002	0.003
aPTT, sec	26.6 (24.5 to 29.1)	27.9 ± 3.2	34.8 ± 9.9	0.0034	0.0042
AT, %	87 ± 16	83 ± 14	61 ± 15	<0.0001	ns
Fibrinogen, mg/dL	234 (197 to 324)	196 ± 52	163 ± 60	<0.0001	0.0001
pH	7.34 (7.29 to 7.37)	7.35 ± 0.07	7.32 ± 0.08	ns	ns
BD, mmol/L	1.9 (0.1 to 3.1)	2.4 (0.8 to 5.2)	5.3 (3.9 to 8.3)	0.0035	0.024
Lactate, mmol/L	2.2 (1.3 to 3.2)	1.8 (1.2 to 3.1)	3.5 (2.3 to 5.5)	0.028	0.014

ISS, injury severity score; Hb, hemoglobin; BD, base deficit; ns, not significant; PT, prothrombin time; aPTT, activated partial thromboplastin time; AT, antithrombin; NCT group, no coagulation therapy; FC group, fibrinogen concentrate only; FC-PCC group, prothrombin complex concentrate and fibrinogen concentrate.

Data are presented as mean ± standard deviation or median (interquartile range). *P*-values are derived from *t*-test or Mann-Whitney *U*-test; significance level *P* <0.05.

ROTEM results upon ER admission are shown in Table 2. There were consistent trends to suggest that the speed of clotting was reduced and clot strength was decreased in the FC group versus the NCT group, and in the FC-PCC group versus the FC group. Differences between the FC-PCC group and the NCT group reached statistical significance.

**Table 2:** ROTEM findings on emergency room admission

	**NCT group (n = 23)**	**FC group (n = 21)**	**FC-PCC group (n = 17)**	***P*-values**	
				**FC-PCC versus NCT**	**FC-PCC versus FC**
**EXTEM**					
CT, sec	58.2 ± 9.5	70.2 ± 21.6	72.6 ± 31.5	0.045	ns
CFT, sec	102.3 ± 29.9	116.0 (96.5 to 160.0)	123.0 (109.0 to 165.0)	0.001	ns
CA10, mm	55.2 ± 6.7	48.1 ± 7.8	46.3 ± 9.2	0.001	ns
**FIBTEM**					
CA10, mm	12.0 (10.0 to 15.0)	8.0 (7.0 to 12.5)	8.0 (4.3 to 11.8)	0.0094	ns

CA10, clot amplitude after 10 minutes running time; CT, clotting time; CFT, clot formation time; EXTEM, extrinsically activated test; FIBTEM, extrinsically activated test plus cytochalasin D; NCT group, no coagulation therapy; FC group, fibrinogen concentrate only; FC-PCC group, prothrombin complex concentrate and fibrinogen concentrate. Data are presented as mean ± standard deviation or median (interquartile range).

*P*-values are derived from *t*-test or Mann-Whitney *U*-test; significance level *P* <0.05; ns, not significant.

**Figure 1:**
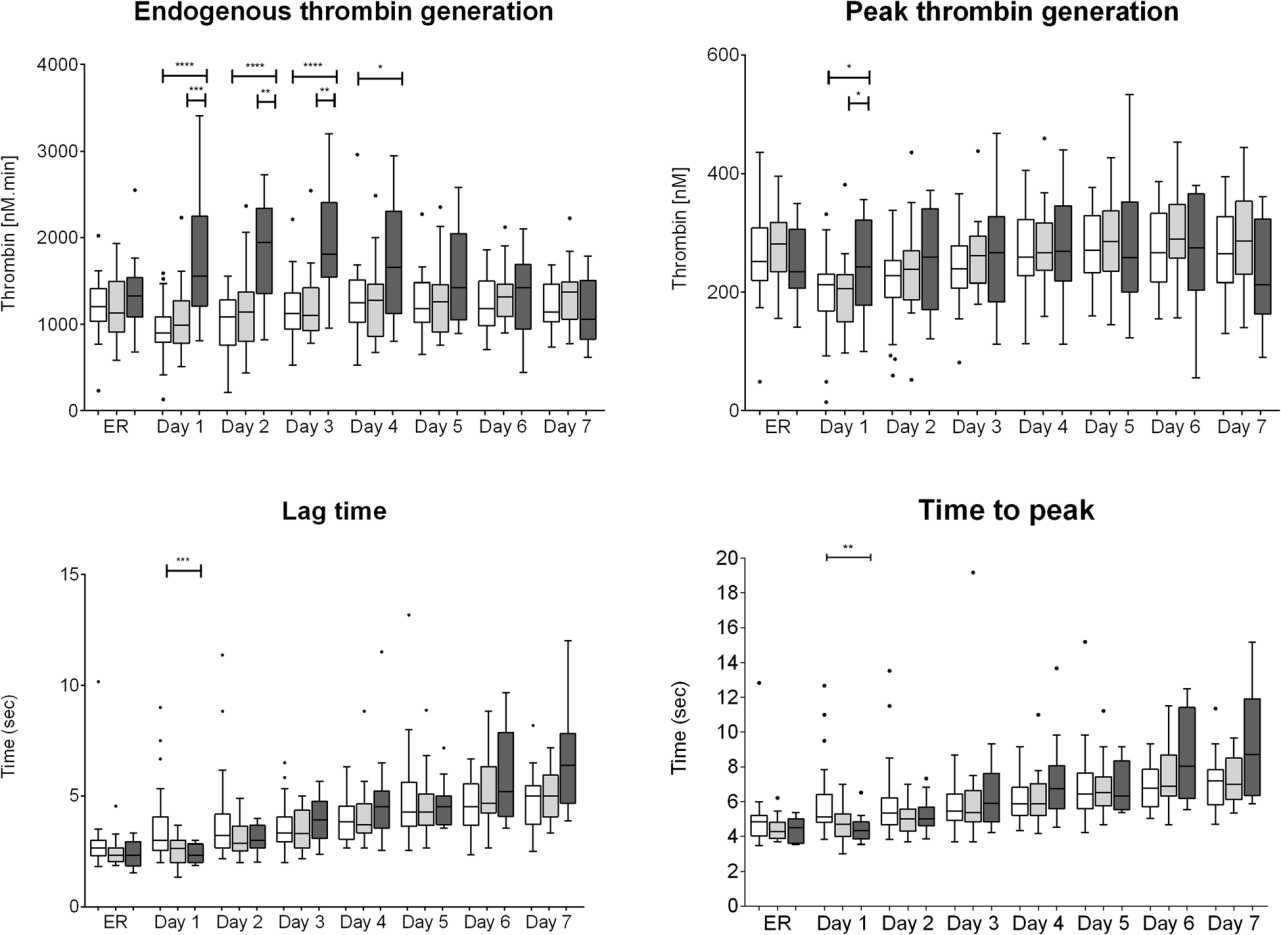
**Parameters relating to thrombin generation, from emergency room (ER) admission until day 7.** Endogenous thrombin potential was significantly higher over the first 3 to 4 days in the prothrombin complex concentrate-fibrinogen concentrate group (FC-PCC group) (dark gray) compared to the fibrinogen concentrate group (FC group) (light gray) and patients receiving no coagulation therapy (NCT group) (white). AUC, area under curve. *P <0.05; **P <0.01; ***P <0.001; ****P <0.0001; no indication = not significant. Data are presented as box and whisker plots (Tukey). Student *t*-test or Mann-Whitney rank sum test was used as appropriate for between-group comparisons.

**Figure 2:**
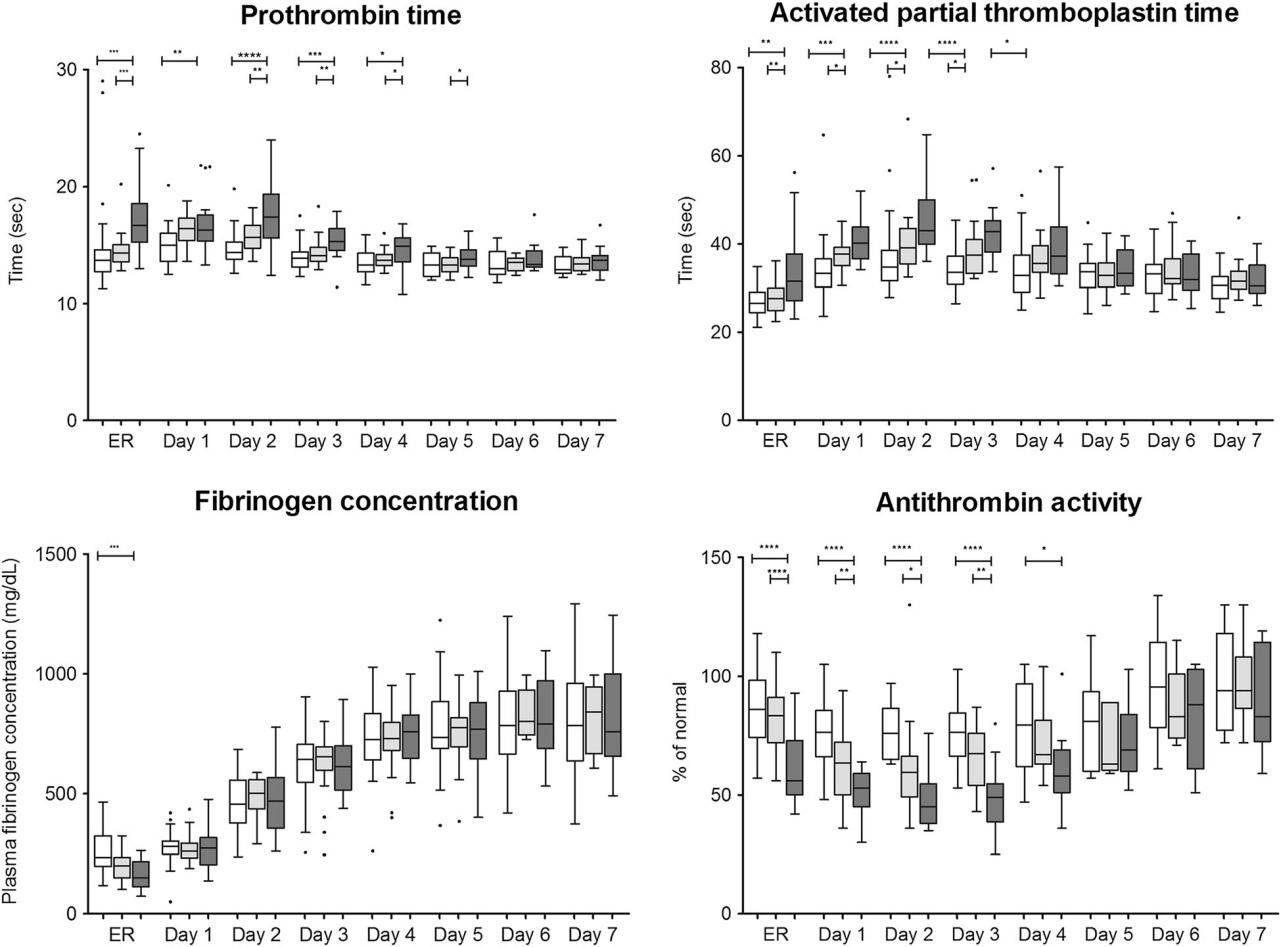
**Prothrombin time, activated partial thromboplastin time, fibrinogen concentration and antithrombin activity from emergency room (ER) admission until day 7.** The prothrombin complex concentrate-fibrinogen concentrate group (FC-PCC group) (dark gray) was compared with the fibrinogen concentrate group (FC group) (light gray) and patients receiving no coagulation therapy (NCT group) (white). *P <0.05; **P <0.01; ***P <0.001; ****P <0.0001; no indication = not significant. Data are presented as box and whisker plots (Tukey). Student *t*-test or Mann-Whitney rank sum test was used as appropriate for between-group comparisons.

**Figure 3:**
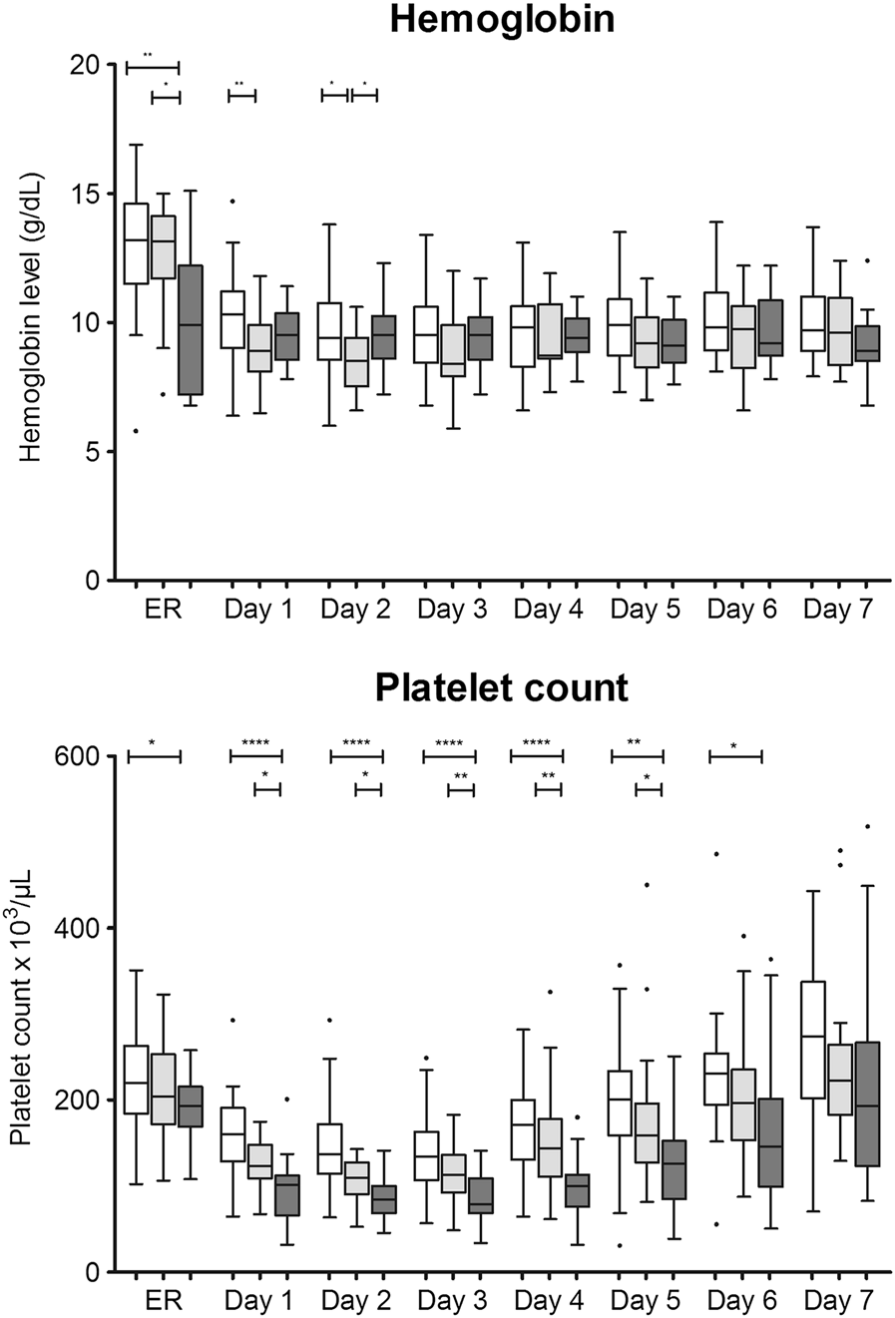
**Hemoglobin and platelet count from emergency room (ER) admission until day 7.** The prothrombin complex concentrate-fibrinogen concentrate group (FC-PCC group) (dark gray) was compared with the fibrinogen concentrate group (FC group) (light gray) and patients receiving no coagulation therapy (NCT group) (white). *P <0.05; **P <0.01; ****P <0.0001; no indication = not significant. Data are presented as box and whisker plots (Tukey). Student *t*-test or Mann-Whitney rank sum test was used as appropriate for between-group comparisons.

**Figure 4:**
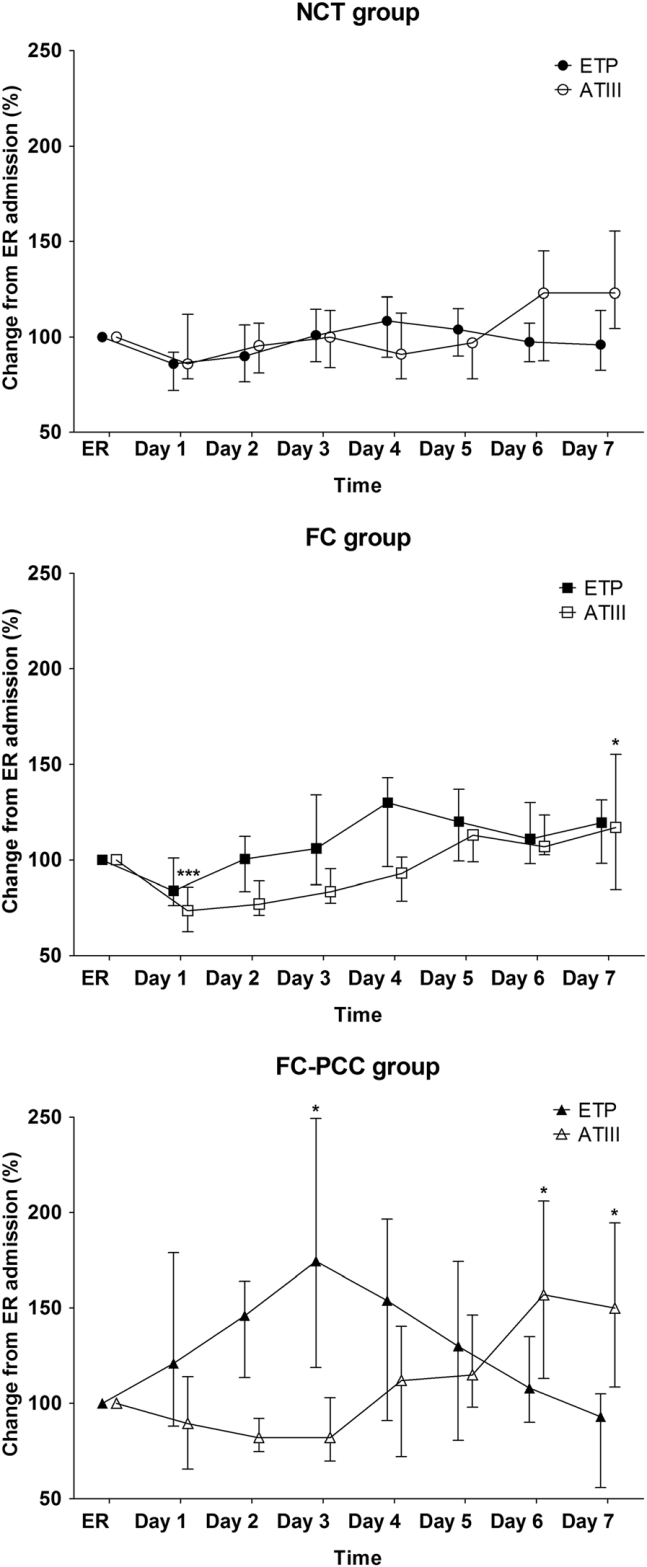
**Relationship between change in endogenous thrombin potential and change in antithrombin, from emergency room (ER) admission until day 7 following trauma.** In the FC-PCC group, a pronounced gap between endogenous thrombin potential and antithrombin was observed. ETP, endogenous thrombin potential (solid symbols); AT, antithrombin (open symbols); NCT group (triangles), patients who received no coagulation therapy; FC group (squares), patients who received fibrinogen concentrate only; FC-PCC group (circles), patients who were treated with prothrombin complex concentrate and human fibrinogen concentrate. *P <0.05; ***P <0.001; not indicated = not significant (within-group comparison versus ER admission). Data are presented as median (interquartile range).

Table 3 provides a summary of hemostatic therapy administered to the three patient groups. The FC-PCC group received a significantly higher dose of fibrinogen concentrate than the FC group (*P* = 0.0002). Significantly more red blood cells and platelet concentrate were transfused in the FC-PCC group than in the FC group and NCT group (both *P* <0.0001). FFP was administered to only one patient in the FC-PCC group.TG-related data are depicted in Figure 1. There were no significant differences between the groups in ETP upon ER admission but subsequently ETP was significantly higher in the FC-PCC group compared with either the NCT group (days 1 to 4) or the FC group (days 1 to 3). The only statistically significant between-group differences in peak thrombin generation were observed on day 1, with a significantly higher value in the FC-PCC group than in either of the other study groups. Similarly, with lag time and time to peak, significant between-group differences were only seen on day 1 (lower values in the FC-PCC group than in the NCT group).Standard coagulation test results are depicted in Figure 2. Significant between-group differences in PT and aPTT were observed upon ER admission and over the following 3 to 4 days. Despite ETP being higher and lag time and time to peak being shorter in the FC-PCC group, PT and aPTT were significantly prolonged in this group compared with either the FC group or the NCT group. This represents an apparent discrepancy between standard laboratory parameters and measurement of thrombin potential.AT upon ER admission was significantly lower in the FC-PCC group than in either the FC group or the NCT group and remained low over the following 3 to 4 days (Figure 2). However, there were no significant between-group differences on days 5 to 7. Figure 2 also shows that the only significant between-group difference in plasma fibrinogen concentration during the study was upon ER admission, when there was a lower value in the FC-PCC group than in the NCT group. Similarly, the only differences between the FC-PCC group and the other groups in hemoglobin were on admission to the ER (low hemoglobin in the FC-PCC group) and day 2 (higher value than the FC group) (Figure 3). Platelet counts were lower in the FC-PCC group at all time points, although there were no statistically significant between-group differences on day 7 (Figure 3).Relative changes in ETP and AT from ER admission until day 7 following trauma are depicted in Figure 4. No clear change over time was evident in ETP or AT among the NCT and FC groups, although there was a suggestion of increased AT levels in the NCT group on days 6 and 7 and an increase over time in both AT and ETP in the FC group. The only statistically significant changes versus ER admission were lower AT on day 1 in the FC group and higher AT on day 7 in the same group. Temporal changes were more prominent in the FC-PCC group: ETP increased continually over the first 3 days to reach a level significantly higher than at ER admission, then returned to baseline over the following 4 days. AT changed little in this group during the first 3 days but subsequently increased to reach levels significantly above baseline on days 6 and 7. A prominent gap between ETP and AT was apparent in the FC-PCC group between days 1 and 4, but not in the other two study groups.

**Table 3:** Blood transfusion and hemostatic therapy during the first 24 hours after hospital admission

	**NCT group**	**FC group**	**FC-PCC group**	***P*-value**
**RBC, U**	0 (0 to 2)	3 (0 to 5)	8 (6 to 10.5)	<0.0001
**FFP, U**	0 (0 to 0)	0 (0 to 0)	0 (0 to 0)	ns
**Platelet concentrate, U**	0 (0 to 0)	0 (0 to 0)	0 (0 to 1)	<0.0001
**Fibrinogen concentrate, g**	0 (0 to 0)	3 (3 to 5)	8 (5 to 11)	0.0002
**PCC, IU**	0 (0 to 0)	0 (0 to 0)	2,400 (1,650 to 2,500)	nc

RBC, red blood cells; FFP, fresh frozen plasma; nc, not calculated; ns, not significant; PCC, prothrombin complex concentrate; NCT group, no coagulation therapy; FC group, fibrinogen concentrate only; FC-PCC group, prothrombin complex concentrate and fibrinogen concentrate. Data are presented as median (interquartile range). *P*-values are derived from the Kruskal-Wallis test; significance level *P* <0.05.

## Discussion

To the best of our knowledge, this is the first study investigating TG-related parameters following PCC administration in acute trauma patients. PCC therapy resulted in significantly higher ETP than in patients who received fibrinogen concentrate only or no coagulation therapy at all and, importantly, this was sustained over the first 3 to 4 days following PCC administration. AT was significantly lower in the FC-PCC group from ER admission until 3 to 4 days later, reaching a nadir on day 2. Hemostasis relies on a delicate balance between pro- and anticoagulant factors, and between thrombin potential and thrombin inhibition potential. This balance may have been impaired in the FC-PCC group, during a period when fibrinogen levels were increased above the normal range; similar findings have been reported in previous studies [34,35]. The overall picture is increased thrombin potential (day 1 to day 4), increased substrate for coagulation (that is, fibrinogen reaching a plateau on day 4) and decreased potential for coagulation inhibition (AT reaching a nadir on day 2). To investigate the potential prothrombotic risk, we recommend that all future studies of PCCs - in all indications including warfarin reversal, new oral anticoagulant reversal and perioperative bleeding - include assessment of thrombin generation for at least 4 days post-treatment.

A well-established indication for PCC in trauma is the rapid reversal of oral anticoagulation by vitamin K antagonists [36,37]. PCC is licensed in Europe more broadly for the treatment or prophylaxis of bleeding in acquired deficiency of prothrombin complex coagulation factors. Small observational studies of PCC in trauma patients suggest that PCC is useful as a component of hemostatic therapy in bleeding patients [13-15,23,25,26,36,38]. The European trauma guidelines recommend PCC primarily for emergency reversal of vitamin K-dependent oral anticoagulants [39]. PCC therapy, guided by thromboelastometric evidence of delayed coagulation initiation, is nevertheless advocated as part of a strategy based on goal-directed administration of coagulation factor concentrates [39]. Outside life-threatening bleeding, PCC should only be administered after fibrinogen supplementation because, as shown previously, the administration of fibrinogen is liable to shorten CT [40].

Importantly, the current study demonstrated poor agreement between TG-related data and standard coagulation tests. Prolonged PT and aPTT suggest deficient TG in the FC-PCC group when the true thrombin potential, as shown by ETP, is increased. Similar findings have been reported previously [27,41]; major shortcomings with PT and aPTT include cessation upon formation of the first fibrin strands when only around 5% of the total thrombin has been generated, and lack of sensitivity to anticoagulants such as AT [12,41,42]. TG is not usually deficient during the early stages of TIC, with evidence suggesting that it is more likely to be increased [12,43,44]. There is a risk that standard coagulation parameters may lead to unnecessary PCC therapy when the true thrombin potential is adequate, potentially increasing the patient’s risk of thromboembolism. The use of standard coagulation tests for guidance of any hemostatic therapy is therefore highly questionable. EXTEM CT, suggested as a means of guiding PCC therapy, also appears not be optimal for this purpose [27]. Improved diagnostic testing, possibly relating to thrombin potential, is needed to guide PCC administration to trauma patients.

ETP is strongly dependent on the concentration of prothrombin (FII), and this has been illustrated by a linear relationship between prothrombin concentration and ETP in prothrombin-deficient plasma [45]. Such correlation was not observed for other coagulation factors such as FV, FVII, FX and FXI. Hanker-Dusel *et al*. investigated the ability of different PCC preparations to generate thrombin in coumadin-treated plasma. The PCC with the highest content of FII produced the largest amount of thrombin [46]. These findings have been confirmed in a rabbit model, where thrombus formation was observed in all animals receiving high amounts of prothrombin [47]. The long half-life of prothrombin (60 to 72 h) [48] means that the effects of PCC therapy may potentially last for a prolonged period of time.

Relative quantities of coagulation factors and inhibitors differ significantly between different PCC preparations [49,50], although the ratio of AT to prothrombin is consistently low [46,49]. AT is the most important inhibitor of thrombin [46,51,52], meaning that physiological ratios between prothrombin and AT are needed for normal levels of TG [46,52]. A substantial increase in FII accompanied by a reduction in AT, as observed in the current study, must be assumed to increase the risk of thromboembolic complications [52]. Labeling of PCCs according to FII content has been proposed [46] but, until this is implemented, physicians are prone to be unaware of the dose of FII with PCCs and the fact that it exceeds the FIX dose [50]. There is therefore a risk of excess FII being administered. The risk may be increased for products with a high FII:FIX ratio or a similar FII:FIX ratio but higher quantity of FIX per vial. The need for caution is underlined by the history of thromboembolic complications with PCC [10], by animal data indicating risks of thromboemboli and DIC [27], and by the fact that another thrombin generation drug, recombinant activated FVII (rFVIIa), has been reported to increase the risk of thromboembolic adverse events among bleeding patients without hemophilia [53]. A recent study showing that PCC may improve TG among neonates undergoing cardiopulmonary bypass also highlighted the need for caution in relation to prothrombotic risk [54].

At a dose of 50 IU/kg, one PCC preparation produced no clinical evidence of thrombosis in healthy volunteers [55]. However, unlike in trauma patients, the thrombin inhibition potential would be normal among both healthy volunteers and warfarin patients. The safety of PCC in trauma patients has not yet been established, and it may be important that the effects upon ETP are variable between PCCs from different manufacturers [49]. In principle, there are two options for controlling thrombin potential following PCC administration. First, AT could be supplemented [56]; Hanker-Dusel *et al*. suggested 1:1 as the optimal administration ratio of AT to FII [46]. The second option is to minimize the administered dose of PCC, to generate the minimum increase in thrombin potential necessary for adequate hemostasis. However, in acute bleeding this is difficult to achieve and routine, rapid estimation of thrombin potential is not currently possible. We have revised our treatment protocol for patients receiving PCC during the acute treatment phase. As a new step, the plasma activity of AT is monitored and, after bleeding is under control, AT is administered if the activity falls below 80% (lower limit of the normal range). Importantly, we do not administer AT during the acute bleeding phase due to the potential risk of aggravating coagulopathy. There is no evidence whether this strategy will reduce the risk of thromboembolic complications. However, the concept of AT monitoring/supplementation in conjunction with PCC has existed for at least 15 years and it has been advocated by the influential Paul Ehrlich Institute in Germany [46,57].

The present study points to an important difference between administration of PCC and fibrinogen concentrate as hemostatic therapy. An increasing body of evidence suggests that the effects of fibrinogen concentrate are short-lived, with no significant effect on fibrinogen levels at 24 h and later [58-60]. In contrast, the effects of PCC on thrombin potential appear to last for 4 days post treatment. This duration of effect is consistent with the approximate 60-hour half-life of FII. Studies involving high-dose fibrinogen concentrate or a high baseline/target fibrinogen concentration have consistently shown the lack of a safety signal [59,61-63]. Conversely, studies of thrombin generating drugs including PCCs have indicated the potential for increased risk of thromboembolic complications [27,46,64]. These data provide a rationale for supplementing fibrinogen first and prothrombin complex coagulation factors second.

### Limitations

This study of hemostatic therapy with PCC lacked a comparator group of patients with the same characteristics (for example, ISS) receiving alternative treatment, such as FFP. Hemostatic therapy was administered in accordance with the criteria documented in the treatment algorithm used at our center [30]. As a result, patients in the different study groups had differing characteristics/severity of coagulopathy, meaning that differences in outcomes may not have been caused solely by the treatments defining the study groups. However, the primary result (significantly higher ETP in the FC-PCC group than in the other patient groups) can be attributed to PCC administration.

Fluid therapy, either in the pre-hospital phase or during the course of initial care, was not evaluated. High-volume fluid therapy results in dilution of both coagulation factors and inhibitors, but not necessarily to the same extent. Thrombin generation has been reported to increase stepwise with increasing hemodilution up to a dilution level of 40%, and to remain above baseline to a dilution level of 80% [43]. This is attributable to a greater decrease in coagulation inhibitors, particularly AT, compared with coagulation factors [43,51]. Assuming that the FC-PCC group comprised patients with more severe bleeding than the other patient groups, larger quantities of fluid are likely to have been administered. The amount of fluid and its impact on thrombin generation among our patients is unclear, but the assumed increased dilution in the FC-PCC group may have contributed to the lower levels of AT.

Hemostatic therapy was administered according to ROTEM findings, in accordance with the treatment algorithm [30]. Unfortunately, because the focus of this study was on thrombin generation, we did not collect ROTEM data immediately before and after treatment. ROTEM data are only available for the ER admission time point, and most hemostatic treatment was administered later. Therefore the ROTEM data presented in Table 2 do not represent the values that triggered treatment with PCC or other hemostatic agents.

Assessment of TG *in vitro* using tissue factor as the activator does not necessarily provide accurate representation of the *in vivo* pattern of TG, because physiological conditions are not represented. Uncertainty in this regard is increased by the fact that ETP was the only thrombin-related parameter demonstrating a significant increase in the FC-PCC group over the first 3 to 4 days - other thrombin-related parameters sowed significant between-group differences only on day 1. We did not measure levels of thrombin-antithrombin complex or prothrombin fragments F1 and F2, and these could have provided further insight into the status *in vivo*.

The lack of clinical endpoints in this study means that the implications of our results for patient outcomes cannot be deduced. We do not routinely screen our patients for deep vein thrombosis, in accordance with American College of Chest Physicians guidelines [65]. Therefore, the data needed to analyze thromboembolic events in the present patient cohort are not available. A study of the effects of PCC administration on thromboembolism among trauma patients is clearly warranted. Considering that thromboembolism is reported as a low-frequency complication of PCC therapy [66], a large sample size would be needed (perhaps ten times the number of patients included in the present study). Furthermore, the post-treatment follow-up period for such a study would be around 4 weeks.

## Conclusion

PCC administration for hemostatic therapy in major trauma patients with bleeding results in a significant increase in ETP, sustained for several days. Postoperative increases in fibrinogen levels were observed in all study groups, while patients receiving PCC therapy had lower levels of AT than those treated solely with fibrinogen concentrate. These findings imply a pro-thrombotic state among PCC recipients but this was not indicated by standard coagulation tests. Randomized controlled trials with clinical endpoints are needed to understand the significance of our results and to evaluate the efficacy and safety of PCC administration for TIC. Before such data become available, we recommend careful use of PCC in this setting: administer PCC only to bleeding patients with obviously severe coagulopathy upon admission or prolonged EXTEM CT after fibrinogen supplementation; adhere to the dose ranges specified in the published algorithm [30]; and monitor and potentially supplement AT among PCC recipients.

## Key messages

• PCC may be useful as haemostatic therapy for patients with bleeding trauma requiring increased thrombin generation

• This study shows that PCC administration according to a concentrate-based treatment algorithm was associated with significantly increased endogenous thrombin potential and lower antithrombin levels than in unmatched controls

• The results imply a pro-thrombotic state among PCC recipients, but this was not evident from standard coagulation test results (PT, aPTT)

• Randomized controlled trials are needed to ascertain the implications of our findings for patient outcomes, and to characterize the safety and efficacy of PCC among patients with trauma-induced bleeding

• Before robust evidence becomes available, we recommend careful use of PCC in this setting

## Abbreviations

aPTT: activated partial thromboplastin time; AT: antithrombin; AUC: area under the curve; CA: clot amplitude; CT: clotting time; ER: emergency room; ETP: endogenous thrombin potential; EXTEM: extrinsically activated test; FC: fibrinogen concentrate (only); FC-PCC: prothrombin complex concentrate and fibrinogen concentrate; FFP: fresh frozen plasma; FIBTEM: extrinsically activated test plus cytochalasin D; GCS: Glasgow coma scale; ISS: injury severity score; NCT: no coagulation therapy; PCC: prothrombin complex concentrate; PPP: platelet-poor plasma; PT: prothrombin time; TG: thrombin generation; TIC: trauma-induced coagulopathy; TXA: tranexamic acid.

## Competing interests

HS has received study grants and speaker’s fees from CSL Behring and Tem International. CJS has received research support from CSL Behring and Tem International. MM has received payment for attending advisory board meetings sponsored by CSL Behring. LK declares no conflict of interest. WV received travel reimbursement from CSL Behring for a study group meeting. The study was funded by the AUVA Trauma Centre Salzburg, Austria and Ludwig Boltzmann Institute of Experimental and Clinical Traumatology, Vienna, Austria. The sponsors had no role in study design, data collection and analysis, decision to publish, or preparation of the manuscript.

## Authors’ contributions

HS and CJS conceived the study, performed data collection, analyzed the data and drafted the manuscript. WV, BS, LK and MM contributed to the study design and drafted the manuscript. All authors have read and approved the final manuscript for publication.
